# Preparing the workforce for telehealth practice: a scoping review

**DOI:** 10.3389/frhs.2026.1807317

**Published:** 2026-03-18

**Authors:** Sharon Brownie, Lauren Parkinson-Zarb, Lindy Cochrane, Antonio Bonacaro, Sameeksha Mudigere, Patrick Broman

**Affiliations:** 1 School of Rural Medicine, Charles Sturt University, NSW, Australia; 2 School of Health Sciences, Swinburne University of Technology, VIC, Australia; 3 Health and Social Practice, Wintec, Waikato, New Zealand; 4 Health Services Research, Peter Macalum Cancer Centre, VIC, Australia; 5 Department of Nursing, University of Melbourne, VIC, Australia; 6 Student and Scholarly Services, University of Melbourne, VIC, Australia; 7 Department of Medicine and Surgery, University of Parma, Parma, Italy; 8 Office of the Provost, Curtin University, WA, Australia

**Keywords:** curriculum: nursing, health professions, telehealth, telenursing, virtual care

## Abstract

This review was undertaken with the aim of locating and mapping telehealth or virtual care curricula or competencies for health professionals in training or practice. The design followed the JBI methodology for scoping reviews and conforms with the requirements of the PRISMA Extension for Scoping Reviews (PRISMA-ScR) checklist. Data sources included Medline (Ovid), Emcare (Ovid), Cumulative Index to Nursing & Allied Health (CINAHL), ERIC (EBSCO) and Scopus plus grey literature databases, customized Google searching, targeted websites, and consultation with content experts in June 2024-Jan 2025. The team members worked independently to extract data. Content analysis was used to map the data, with the use of a data extraction table developed by the review team for the purpose of preparing, organising, and reporting identified evidence. Team meetings were used to collate analysis and facilitated consensus regarding the creation of categories and subsequent themes. The search identified 3,189 peer-reviewed works and 7 grey literature items. After screening 31 publications met criteria for inclusion. Analys of included evidence indicated that telehealth services are safe, effective and increase health service access if delivered by appropriately qualified personnel but competence in face-to-face care provision does not automatically transfer to competency in virtual delivery. The search identified a series of competencies pertinent to telehealth-based clinical encounters, including the call management competencies to prepare for a consult; communicate with clients; conduct the consult; and close. The findings provide information in respect to telehealth essential inclusions in undergraduate nursing curricula and nursing professional development programs. The literature demonstrates a slow uptake in embedding new practice models in education programs for the future health and nursing workforce. A need exists to confirm the competency requirements for telehealth delivery and accelerate related content into nursing and health workforce curricula and professional development activities.

## Introduction

Models of virtual care are increasingly being adopted to better utilize expertise across the health workforce, helping to address issues of health service access, particularly for rural, remote, and marginalized communities. Though existing well before the pandemic (1, 2), Covid-19 accelerated virtual care utilization in response to the urgent need for continued care, particularly for those unable to access traditional service while under stay-at-home orders and during community lockdowns (2, 3). Post-pandemic, virtual care services have continued to expand, reflected globally in patient expectations, health service delivery and clinical practice (4). The American Telemedicine Association (ATA) now estimate that by 2030 more than 50% of all healthcare services will be delivered virtually (5).

Virtual care is a safe and effective modality if delivered by appropriately qualified personnel, however, competence in face-to-face care provision does not automatically transfer to competency in virtual care delivery (5, 6). During virtual care encounters the clinician cannot use all senses. The sense of ‘touch’ and ’smell’ is absent in video-based encounters; telephone-based consults are further limited by the absence of sight. This fundamentally different mode of practice thus requires education frameworks to support competency and safe practice (7).

### Background literature

**Figure 1:**
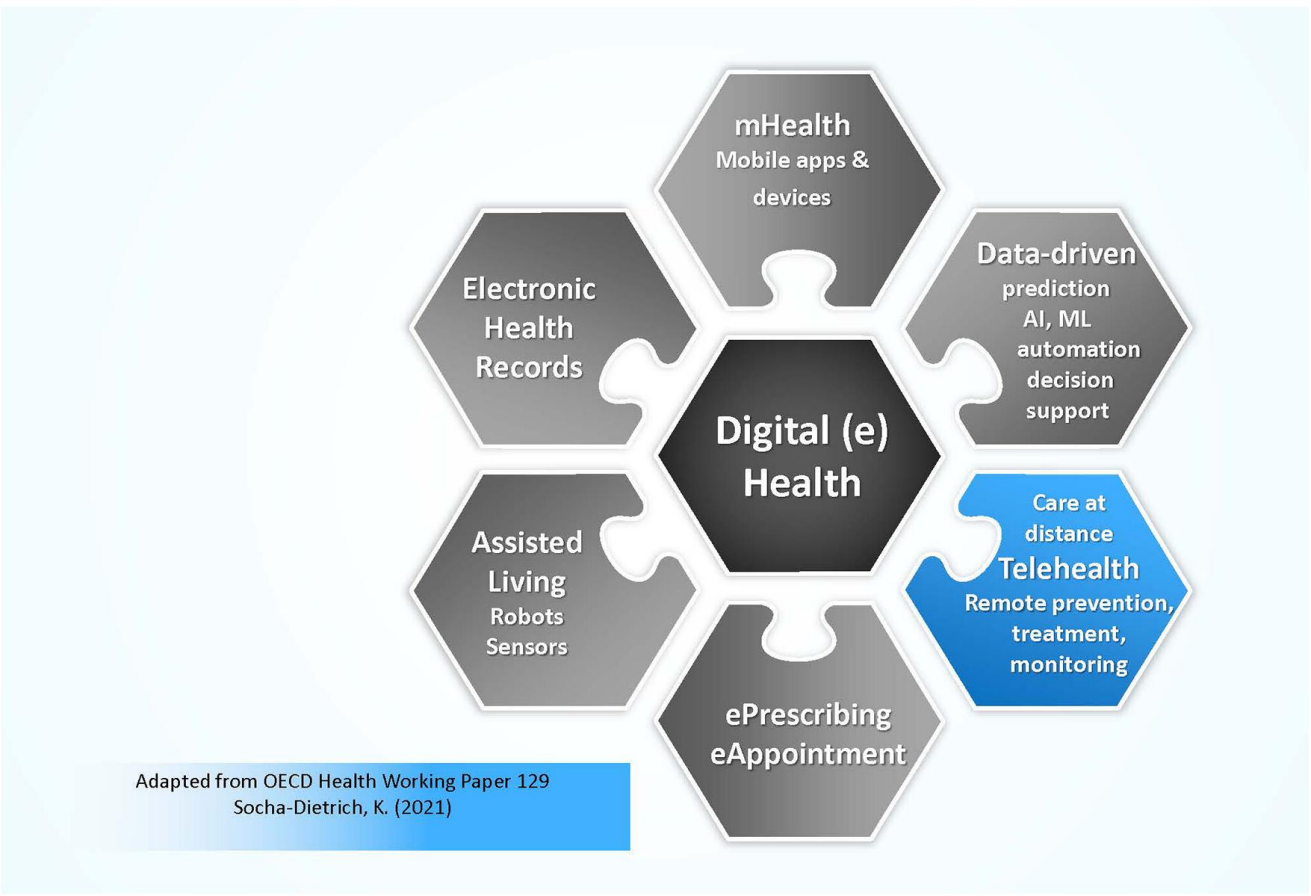
Telehealth as a subset of digital health.

The most consistently used term for virtual care is telehealth, so this term is used henceforth. Telehealth (a subset of digital health or eHealth) includes the use of various information, assessment and communication technologies to provide care in situations where nurses or other health professionals and patients are not physically located in the same place (8, 9). Telehealth encounters may be telephone-based or video-based. In comparison, digital health is a broader term referring the use of information and communication technologies (ICT) to deliver health and nursing related services. Reference to digital health can involve a wide-ranging use of tools, including mHealth, mobile health apps, electronic referrals, electronic prescriptions, wearable devices, electronic records, patient reported symptom monitoring, telehealth modalities and more (9, 10). This search relates specifically to telehealth (see Figure 1) rather than the broader concept of digital health or eHealth.

Telehealth models of care have been utilized for several decades (11); however, the literature portrays a slow uptake in embedding these new practice models in healthcare, particularly nursing education and practice (12). A broad exploration of the literature highlights that more education is needed for identification and acquisition of specific telehealth nursing competencies (13, 14). This is not a nursing issue alone, with telehealth-related education practice gaps reported across medicine and allied health. A major NHS (National Health Service) study across the UK concluded that allied health professionals are not adequately equipped with the skills needed to deliver telehealth, and that they lack access to the guidelines and education to help them do so (15). Similar challenges have been reported for physician assistants (16) and training medical students (17). Given the universality of the education/practice gap across all health professions, literature pertaining to all healthcare professions were included in this review.

Despite years of recommendations, and escalating concerns from scholars that the growth of telehealth has occurred without consideration of necessary education and competency development (6), significant disconnect continues. Telehealth-related content is notably absent from nursing accreditation standards, nursing scopes of practice and undergraduate nursing curricula (18).

This education-practice gap can be traced back over a 45-year period, highlighting the longstanding issue of technology moving faster than education. Early descriptions of the use of the telephone as a tool in the delivery of primary care notes that few if any educational providers had programs for telephone skills development (19). Researchers have stressed the need for educational preparation as an essential component of nurse-led telephone services (19, 20) along with need for modifying existing curricular and standards of practice to ensure that students were prepared for competent delivery of telephone consultation skills (20, 21).

By the turn of the century, authors noted that telephone consultation was now well established in practice and was now an ‘integral’ feature of patient centred care (22). Goodwin asserted that as telephone services continued to proliferate, the nursing profession must be active in defining telehealth practice and establishing a body of knowledge specific to nursing (23). Kamei recommended that education for nurses should include telecommunications training; familiarity with communication devices; and clinical skills education to enhance telenursing competencies (24).

Preparing nurses (and other health professionals) for roles in telehealth delivery is increasingly urgent. It is projected that by 2030 more than half of all health consults will occur virtually (5). Rutledge and Gustin highlight survey results suggesting that more than 25% of health users would change providers to have telehealth options (5). Given these trends, it is critical that educational providers prepare nurses for this telehealth future (5, 25). Nurses now play a pivotal role in facilitating and delivering telehealth services, but nursing education has been slow in responding and most programs do not prepare nurses for telehealth practice (18). Graduates do not commence work with the required telehealth skills and competencies: quality telehealth encounters need effective education and training. It is the responsibility of educational providers to ensure innovations in service delivery are incorporated into undergraduate and continuing education curricula (18, 26).

Exploration of recent studies reveals that many nurses in telehealth practice have had no formal training (27); nursing academics are reported as having failed in the job of preparing nursing graduates for roles in telehealth (5); experience and content across the various levels of nursing education programs appears low (12); and graduates are entering the health workforce without necessary telehealth practice competencies (18). An Australian national study funded by the Victorian Higher Education State Investment Fund reported 69.9% of undergraduate nursing (entry to practice) programs as having no digital health content. The question here is why nursing education has been so slow to respond.

In addition to the education-practice gaps, practice-regulatory gaps are also evident. An example of this disconnect can be seen in the recently revised New Zealand Nursing Council Nursing Education Standards for programs leading to registration as a registered of enrolled nurse (28). The revised standards provide generalized reference to the need for teaching and learning to reflect contemporary practices in digital health systems, but further clarification regarding implications for practice and public safety is absent. Specifically, no definition of digital health is included and no reference made to competencies required for safe practice during clinical encounters in telehealth or virtual care. The standards lack competency descriptors and measures for safe clinical practice in virtual care including safe telephone-based practice.

### Aim

Given the urgent imperative to close the current education-practice gap, this review sought to locate and map telehealth or virtual care curricula or competencies of relevance to nurses in education or practice. Research questions focused the review as follows:

1. What curricula course content, learning outcomes and assessment tools exist of relevance to the development of telehealth related education programs for licensed health professionals, and nurses in particular?
2. What competencies are applicable to safe practice in telehealth assessment and practice across the health workforce, particularly those competencies related to clinical encounters?
3. How can the results of the search be used to inform nursing education?

## Methods

The scoping review was designed to locate studies within both the peer-reviewed and grey literature. Search processes were conducted in accordance with the JBI methodology for scoping reviews (29) and reported in adherence to PRISMA Extension for Scoping Reviews (30).

The search strategy was developed to include both published and unpublished studies. A preliminary search was performed in Medline (Ovid) to identify relevant key terms, subject headings, and indexing specifically associated with telehealth, health assessment, and educational concepts. Additional key terms were identified through a thorough review of the references and citations within relevant studies, and the final search strategy was adapted and applied to Medline (Ovid), Emcare (Ovid), Cumulative Index to Nursing and Allied Health (CINAHL), ERIC (EBSCO) and Scopus, with searches limited to English language papers (see Supplementary Material A1).

Additional searches for grey literature involved exploring grey literature databases, utilising specialized Google searches, visiting targeted websites, and consulting with subject matter experts (31).

### Inclusion and exclusion criteria

Table 1 details the criteria for search inclusion and exclusion. Telehealth origins are traceable 150 years, with an 1879 Lancet publication which recommending its use to avoid unnecessary visits to the office (11). Because of the extensive history related to the evolution of telehealth, only studies within a 30-year time span 1994–2024 are included. This timespan provides visibility and tracking of initial telehealth use followed by the technological innovations leading to the introduction of video-based consults and recommendations for nursing and health professional education.

**Table 1:** Inclusion and exclusion criteria.

Criteria	Inclusions	Exclusions
Population	All health professions Nursing	Non-regulated healthcare workers Specialty medical practice
Telehealth curriculum and competencies	Curriculum studies and/or guidelines for telehealth care Competency focused studies and/or descriptors for telehealth care	Studies related to face-to-face delivery of care Technical guidelines Digital guidelines Incomplete studies Conference abstracts
Comparator and context	All geographies with web -based infrastructure for telehealth care All educational providers (Public, Private, NGO)	Geographies without web -based infrastructure for telehealth care
Study characteristics	Empirical research Case studies Grey literature Quality improvement initiatives	Studies which do not provide guidelines for virtual health assessment
Language	Studies published in English	Studies in other languages
Year of publication	Studies published within the last 30 years (1994–2024)	Studies older than 30 years unless historically significant

In line with research questions, inclusion criteria sought work related to all health professions on the basis that scoping reviews characteristically utilize a wide net to explore literature within a topic of publication paucity. The broad literature is then analysed to focus findings more narrowly and inform further research (32).

### Data extraction and analysis

Content analysis was employed to capture frequency counts of concepts, populations, characteristics (33, 34). This approach was undertaken in accordance with JBI recommendations for reporting of scoping review results while confirming with the requirements of the PRISMA Extension for Scoping Reviews (PRISMA-ScR) checklist (30, 34). The framework recorded details inclusive of names of the authoring team, year of publication, professional grouping, location of study, publication type and the type of guidance provided and key findings of relevance to the review question/s. The final data extraction table is included as Table 2. Initial data extraction was undertaken independently by two reviewers (AB & SM). After further discussion with research team members (PB & LZ), adjustment was made to the organisation of extracted data and descriptors, based on team consensus.

**Table 2:** Extracted studies.

Author and year	Region	Target audience	Publication type	Description of educational intervention and/or competencies	Program delivery	Professional endorsement
Crouch et al. 1997 (20)	UK	Professional development for nurses	Peer-reviewed	Face–to-face programme teaching telephone consultation skills for nurses, including topics such as questioning, listening, advice giving, dealing with difficult callers, and documentation.	Not specified; the details of the programme details are described in separate articles (‘Ringing the changes: developing, piloting and evaluating a telephone advice system in accident and emergency and general practice settings’) which are not available	No
Wilkinson et al. 2000 (36)	North America	Professional development for nurses performing telephone triage	Grey Literature	Describes (including development process) a competency-based curriculum for a telephone triage	Not specified	No
Bishop et al. 2013 (37)	UK	Professional development for physiotherapists	Peer-reviewed	Training in telehealth competencies for providing physiotherapy-led telephone assessment and advice services, including clinical reasoning, communication skills, and patient management	Initial training program (face-to-face for 1 and half days) + ongoing skill consolidation over the telephone	No
Sands et al. 2013 (38)	Australia	Professional development for nurses	Peer-reviewed	Articulates key role tasks, skills, knowledge and responsibilities in which clinicians are required to be competent to perform mental health triage telephone assessment.	NA	No
Johnson et al. 2015	Europe	Professional development for nurses	Peer-reviewed	A formative self-assessment tool to be used by telenurses to become aware of/evaluate unique telehealth communication and interpersonal competencies by analysing their own conversations with patients/callers.	NA	No
Van Houwelingen et al. 2016 (1)	Europe	Training for nurses	Peer-reviewed	Defines set of 14 nursing telehealth entrustable professional activities (NT-EPAs) and 52 competencies required for nursing telehealth activities, which can contribute to development of nursing telehealth education.	NA	No
Butler-Carroll and Philips 2018 (60)	North America	Formal qualification for a range of health professionals	Grey Literature	Outlines a formal certificate in telemedicine. The curriculum features educational games, equipment demonstration, hands-on practice, billing/coding and legal information, and Objective Structured Clinical Examinations (OSCEs) in a simulated clinic setting.	Online modules + hands-on simulated learning (total approx. 40 hours)	No
Hilty et al. 2018 (39)	North America	Professional development for psychiatrists’ and other mental health professionals	Peer-reviewed	Defines competencies for telepsychiatry.	NA	No
Hilty et al. 2019 (57)	North America	Training for health professionals	Peer-reviewed	Offers broad guidance on education for mobile health, smartphones and apps to compliment telehealth encounters and includes competencies, training methods, and faculty development recommendations.	Blended (seminar, case based and problem-based). Varied duration	No
Lum et al. 2020 (43)	Asia	Course content for medical students and residents	Peer-reviewed	A competency-based telehealth consultation training program suitable for final-year medical students and medical residents, which could be adapted for other health professionals. The workshop comprised case discussions and providing participants with feedback on how to improve consultation skills based on prerecorded telehealth conversations.	Half-day front lectures (totalling 5–6 h including a 30 min tea break)	No
Wong et al. 2020 (44)	North America	Course content for medical residents	Peer-reviewed	A simulation activity with online Standardized Patient to teach telehealth competencies, interdisciplinary telemedicine and e-communication skills to first- and third-year medical residents. Elements of how to evaluate skills post the simulation are detailed.	Blended (online and onsite simulation) longitudinal program (Two 120 min training sessions over two weeks (totalling 4 h) plus inter-visit online simulation activities	No
Association of American Medical Colleges 2021 (50)	North America	Medical educators and clinicians	Grey Literature	Describes telehealth competencies for the purposes of medical education. Three developmental stages for each competency are outlined.	NA	Yes
Cornes et al. 2021 (45)	North America	Course content for medical students	Peer-reviewed	Foundational telemedicine workshop for first-year medical students including use of discrete, time-limited activities (self-assessment, templated group exercises, review of brief multimedia, and active role-play).	Single online workshop (approx. 2.5 h)	No
Frankl et al. 2021 (46)	North America	Course content for medical students	Peer-reviewed	Telehealth curriculum for medical students, covering topics such as telemedicine concepts, best practices for setting up visits, conducting history and physical exams, and ethical considerations through webinar-style presentations, prerecorded videos of physical examinations from different disciplines, shadowing a synchronous telemedicine visit, peer discussions in small groups, and quizzes.	5 online modules primarily asynchronous curriculum (approx. 10 h total)	No
George et al. 2021 (26)	North America	Course content for Family Nurse Practitioner and Bachelor of Nursing students	Peer-reviewed	Online telehealth objective structured clinical evaluations in FNP and BSN nursing education. Includes telehealth OSCE, each with participant roles.	Online, length not specified	No
Parish et al. 2021 (40)	North America	Professional development for psychiatrists	Peer-reviewed	Training model for Asynchronous Telepsychiatry (ATP) clinician skills Provides recommendations on skill sets and technology training requirements for clinicians conducting asynchronous telepsychiatry interviews.	Blended (seminar, case supervision, and case discussions, a training manual and one-on-one sessions were combined for initial training.) totalling 75 h (approx. 15 h per interviewer)	No
Quinlin et al. 2021 (54)	North America	Course content for Family Nurse Practitioner students	Peer-reviewed	Objective structured clinical examination evaluation (OSCE) using web conferencing to assess Nurse Practitioners’ telehealth assessment skills. Includes assessment guide for awarding marks with extensive detail provided about elements within each domain. Small groups were debriefed online.	Single online session (approx. 2 h and half)	Yes
Bobek 2022 (55)	North America	Educators teaching Nurse Practitioners	Peer-reviewed	Teaching strategies for telehealth competencies, including specific techniques, resources, and educational strategies.	Program length may vary depending on the context, lasting from 8 h to 2 weeks, depending on setting	No
Health Education and Training Institute, NSW (HETI) 2022 (58)	Australia	Professional development for a range of health professionals	Grey Literature	An educational framework for virtual care education implementation, including various evidence-based telehealth domains and competencies.	Not specified	Yes
Health Education and Training Institute, NSW (HETI) 2022 (59)	Australia	Professional development for a range of health professionals	Grey Literature	Outlines curriculum interventions and pedagogical approaches for virtual care, including curricula enactment phases and key content. Values, behaviours and skills required to deliver virtual care are summarised.	Not specified	Yes
Kumra et al. 2022 (47)	North America	Course content for medical students	Peer-reviewed	Outlines method of self-efficacy assessment for telemedicine clinical skills in second year medical students, via simulated telemedicine case in the form of a Standardised Patient.	Single online session (15 min)	No
Liew et al. 2022	North America	Course content for medical and Physician Assistant students	Peer-reviewed	Telepsychiatry workshop for teaching telemedicine safety competencies for suicide risk assessment; a combination of lectures, participatory exercises, and simulations. Participants included third-year medical students and second-year PA students. Includes details on curriculum, activities, and assessment methods.	Single online session of 4 h and 45 min.	No
Sheikh 2022 (56)	North America	Course content for Nurse Practitioner students	Peer-reviewed	Provides guidance on telehealth simulation, as part of a multimodal approach for nurse practitioner training. Details learner-centred approach of rapid-cycle deliberate practice (RCDP) as integrative learning strategy.	Multiple sessions, in-person and online, over 10 weeks (approx. 20 h total	Yes
Venditti et al. 2022 (49)	North America	Training for medical residents	Peer-reviewed	Reports learner and faculty perceptions of telemedicine training for family medicine residents. Competency, domains and key telehealth skills are summarised.	NA	No
Bajra et al. 2023 (51)	North America	Course content for medical students	Peer-reviewed	Program and learning outcomes of an online workshop for telehealth skills curriculum for clerkship-level medical students based on the Association of American Medical Colleges (AAMC) telehealth competencies. Criteria for an OSCE to assess learners’ application of knowledge is presented. The modules incorporated instructional videos, animations, and interactive exercises to foster effective learning.	5 self-paced modules over 4 months (approx. 20 h total)	Yes
Bajra et al. 2023	North America	Course content for medical students and residents	Peer-reviewed	Outlines a telemedicine curriculum focused on providing medical students and family medicine residents with the knowledge, skills, and attitudes necessary to effectively integrate telemedicine into practice. I agree	Blended: Virtual telehealth workshop (80 min) + clinical encounters + formative tele-OSCEs during last week of clerkship.	Yes
Liew et al. 2023 (48)	Asia	Course content for medical students	Peer-reviewed	A simulation-based training module on virtual consultations with standard patients, asynchronous lessons, videos and reflective reports in year 4 and 5 medical students and in newly graduates.	6-week module (approx. 30 hours total), blended (online microlearning and simulation-based)	No
McConnell et al. 2023 (52)	North America	Course content for medical residents	Peer-reviewed	Telehealth Curriculum for Internal Medicine Residents Featuring a Virtual Physical Examination, Details competencies for telehealth legal guidelines; virtual physical examination; health equity; telehealth chronic disease management; demonstrations, handouts, and verbal coaching on adapting physical examinations to a virtual setting; case-based scenarios simulation.	3 synchronous online sessions (approx. 6 h total)	No
Moore and Bonefant 2023 (41)	North America	Professional development for oncology	Grey Literature	An in-person or virtual education program for oncology nurses telephone triage, focusing on 1) how to manage symptoms associated with the oncology patient’s diagnosis and treatment plan; 2) to increase the nurse's knowledge regarding how to educate, advocate, and provide a plan for patients over the telephone; and 3) to support learner's transition to telephone triage.	8 h training session (in-person or virtual).	Yes
Palesy et al. 2023 (53)	North America	Training for medical students	Peer-reviewed	Curriculum Interventions and Pedagogical Approaches for Virtual Care Delivery. Virtual Care curricula, enactment phases and key content are summarised.	NA	No
Grosjean et al. 2024 (42)	Europe	Professional development for health professionals	Peer-reviewed	Educational guidance on digital health including a skills framework, training modules, and teaching resources for digital health education.	Blended online and face-to-face 5-year project with various modules (approx. 20–30 min each)	No

Quality appraisal was not undertaken, as our intention was to locate and describe the current telehealth educational competencies currently available, not to analyse the quality of the included evidence (35). A team approach was maintained throughout the process of data extraction, analysis, and presentation, to ensure regular robust discussion of any issues encountered.

## Results

**Figure 2:**
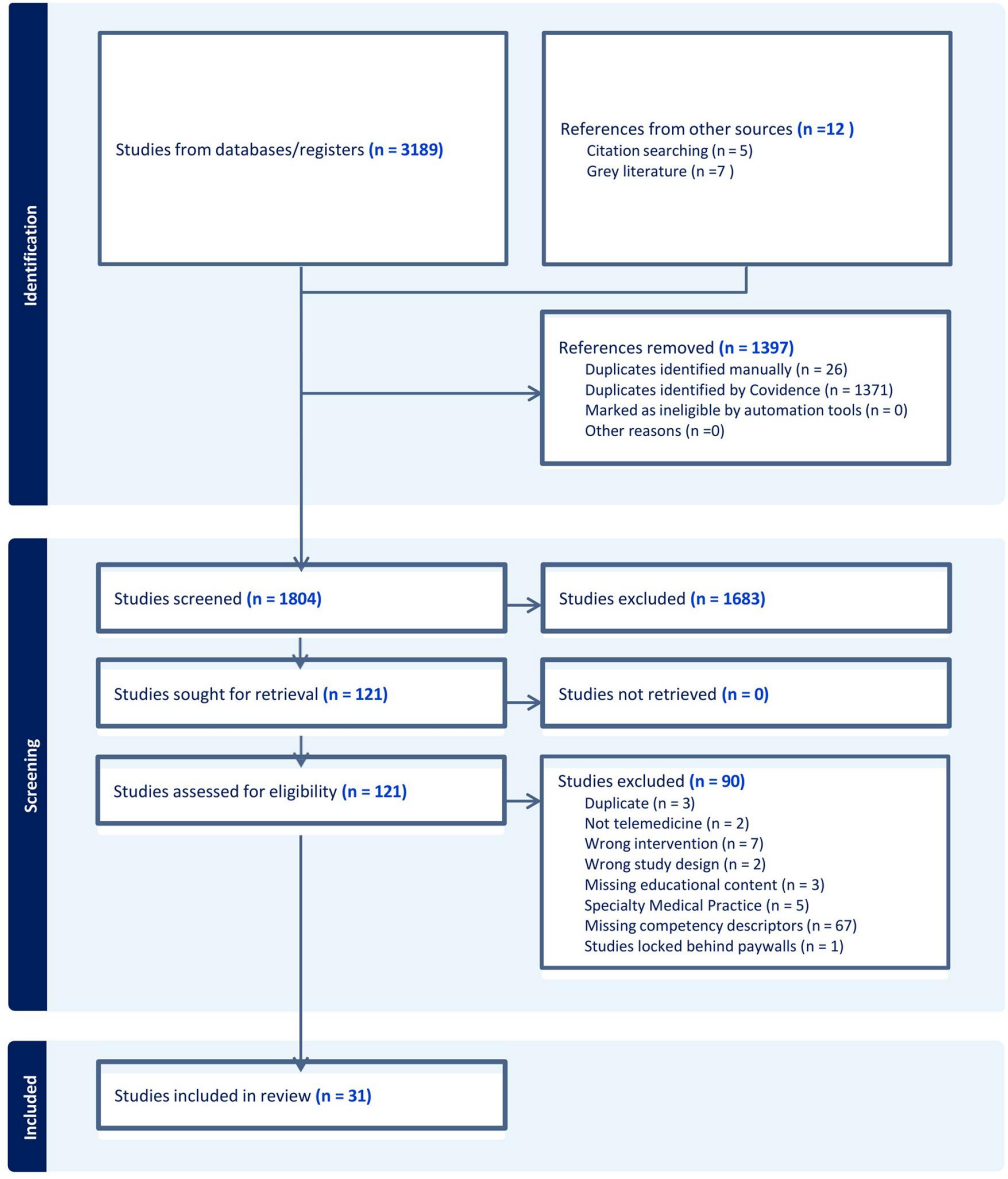
PRISMA flow diagram of selected articles.

The literature search was commenced in June 2024, concluded in Jan 2025 and identified 3,189 peer-reviewed works and 7 grey literature items. After screening 31 studies met the eligibility criteria for inclusion (Figure 2).

### Characteristics of evidence sources

Twenty-five publications were drawn from the peer-reviewed literature with six derived from grey literature sources. The spread across professions involved medicine *n* = 13, nursing *n* = 10, health professional teams *n* = 5, mental health professionals *n* = 2, and allied health (physiotherapy) *n* = 1.

While earlier published studies (20, 36) discussed competencies for telephone-based services, the first study explicitly referring to video-based delivery was van Houwelingen et al. (2016), with mobile health, smartphones, and apps first mentioned in Hilty et al. (2019). The number of located works increased markedly from 2018 onwards with only 7 of the 31 manuscripts published before this date, paralleling increases in telehealth practice since that time.

Publications in this area primarily described short professional development activities for the existing workforce rather than inclusions within health professional curricula (20, 36–42). The medical profession stood out as most active in developing components of telehealth content and competency development within existing programs - undergraduate to registrar (43–53) and works specific to psychiatry and mental health (40, 48). Bajra et al. (2023) go a step further by describing development of a national curriculum for embedding in medical programs.

Nursing related works focussed primarily on nurses in nurse practitioner level roles (26, 54–56) or nurses working in a speciality field such as oncology (41). Allied health, specifically, physiotherapy featured in one study only (37). The works pertaining to health professional teams was noted in peer reviewed literature (39, 57) and grey literature (58–60) with one describing activities related to a formal qualification (Certificate in Telemedicine) inclusive of assessments involving formal OSCE examination as confirmation of competency (60).

### Identification of competency descriptors

Following exaction of the data detailed in Table 2, further content analysis was undertaken to identify the competencies described within each work. Analysis was undertaken by three members of the review team (PB, AB & SM) using an *a priori* framework developed with consensus of the full team and based close reading of all works. Such an approach provided guidance for the three team members to locate and record competencies and curricula content related to preparation for telehealth consultations; telehealth related communication, the telehealth consult; and actions after the consult (see Table 3).

**Table 3:** Identified telehealth competency descriptors and curricula content.

Author and year	Preparation for telehealth consultation			Communication			The consult				After the consult			Discharge
	Equipment familiarization	Compliance: ethics, safety, confidentiality	Review patient profile	Building rapport. culture & context	Questioning, explaining & information Sharing	Complexity e.g., silent or abusive clients, language, barriers interpreter	History	Triage	Assessment	Client & family education next steps	Debrief/referral	Escalation of care	Admin Notes/Next APPT	
Crouch et al. 1997 (20)	✓	✓	✓				✓	✓			✓	✓		✓
Wilkinson et al. 2000 (36)	✓	✓	✓	✓			✓	✓	✓	✓				
Bishop et al. 2013 (37)	✓						✓							
Sands et al. 2013 (38)	✓	✓	✓	✓	✓	✓	✓	✓	✓	✓	✓			✓
Johnson et al. 2015					✓		✓		✓			✓		✓
Van Houwelingen et al. 2016 (1)		✓	✓			✓	✓				✓			✓
Butler- Carroll and Phillips 2018 (60)	✓		✓				✓	✓		✓	✓	✓		✓
Hilty et al. 2018 (39)	✓	✓	✓			✓	✓	✓	✓	✓	✓			✓
Hilty et al. 2019 (57)	✓	✓	✓			✓	✓	✓	✓	✓	✓			✓
Lum et al. 2020 (43)	✓	✓	✓	✓	✓	✓	✓	✓	✓	✓	✓			✓
Wong et al. 2020 (44)			✓			✓				✓	✓		✓	
Association of American Medical Colleges 2021 (50)	✓	✓	✓	✓	✓	✓	✓	✓	✓	✓	✓	✓		✓
Cornes et al. 2021 (45)														
Frankl et al. 2021 (46)		✓	✓		✓		✓	✓						
George et a. 2021 (26)	✓	✓			✓	✓				✓	✓			✓
Parish et a. 2021 (40)	✓	✓	✓				✓		✓			✓		✓
Quinlin et al. 2021 (54)			✓			✓	✓	✓	✓		✓	✓	✓	
Bobek 2022 (55)		✓					✓				✓		✓	
Health Education and Training Institute (HETI) 2022 (58)	✓	✓	✓	✓	✓	✓	✓	✓	✓	✓	✓		✓	✓
Health Education and Training NSW (HETI) 2022 (59)	✓	✓	✓			✓	✓	✓	✓	✓	✓	✓	✓	✓
Kumra et al. 2022 (47)	✓	✓	✓		✓	✓	✓	✓	✓			✓		✓
Liew et al, 2022	✓	✓	✓		✓	✓	✓	✓	✓	✓	✓	✓	✓	✓
Sheikh 2022 (56)						✓	✓				✓		✓	✓
Venditti et al. 2022 (49)	✓	✓	✓		✓	✓	✓	✓	✓	✓	✓			✓
Bajra et al. 2023 (51)	✓	✓	✓	✓	✓	✓	✓	✓	✓	✓	✓	✓		✓
Bajra et al, 2023	✓		✓	✓		✓	✓		✓				✓	
Liew et al. 2023 (48)	✓	✓	✓	✓	✓	✓	✓	✓	✓	✓	✓	✓	✓	✓
McConnell et al. 2023 (52)		✓					✓		✓			✓		✓
Moore and Bonefant 2023 (41)	✓	✓	✓	✓	✓	✓	✓	✓	✓	✓	✓	✓		✓
Palesy et al. 2023 (53)	✓	✓	✓	✓	✓	✓	✓	✓	✓	✓	✓	✓		✓
Grosjean et al. 2024 (42)	✓	✓	✓	✓	✓	✓	✓	✓	✓	✓	✓	✓	✓	✓

**Figure 3:**
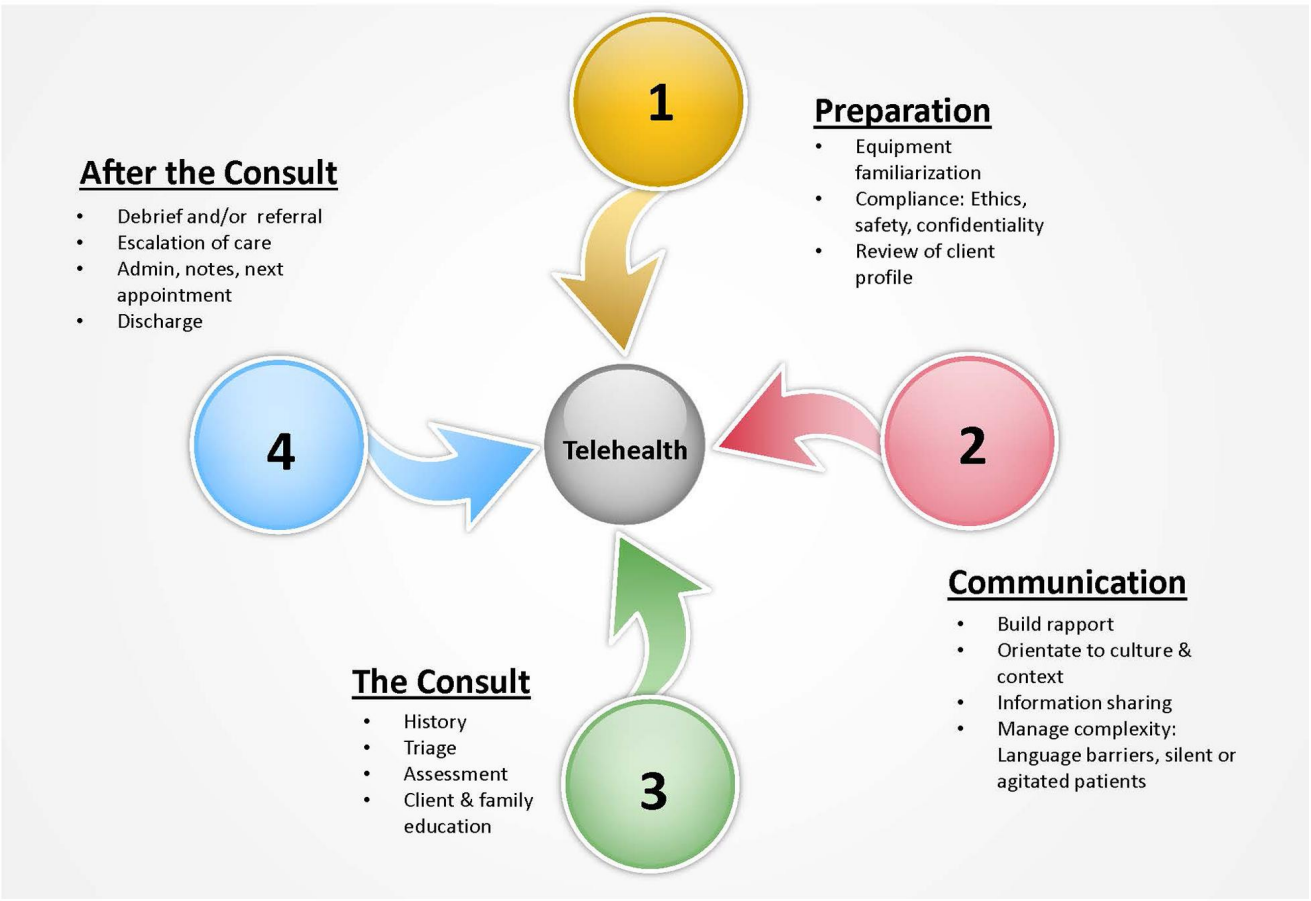
Call management steps and competency descriptors required for telehealth practice.

These three team members (PB, AB & SM) independently located the competency descriptors and/or curricula content related to telehealth practice within each of the articles. Reviewers then coded them within each of the four overarching categories and sub-categories in Table 3. The categories and sub-categories were developed following data analysis identifying patterns across clinical call encounters described within the selected articles. A group meeting was then convened to resolve any conflicts between the three reviewers. Consensus of the competency categories and sub-categories was then used to develop an illustrative portrayal of findings as shown in Figure 3.

## Discussion

This review was undertaken with the aim of locating and mapping existing telehealth curricula or competencies for all health professionals in training or practice and then considering these in relation to their relevance for nursing practice. Review questions focussed on three areas, specifically, 1) the presence of telehealth related curricula and assessment material within existing peer-reviewed and grey literature, 2) competencies applicable to safe practice in telehealth delivery for all health professionals but nurses, and 3) the implications for nursing education.

### Research question 1: existing telehealth curricula

Telehealth is now core business transforming health systems beyond COVID-19 crisis mode responses, changing the full spectrum of patient care (61–64). Despite these developments, this review failed to identify any reported example of telehealth related curricula content fully embedded as complete course modules within accredited undergraduate or post graduate nursing or heath professional programs. Subsequently, this discussion focusses on the pockets of development identified in this search along with gaps requiring further focus.

A recent French initiative offers a partial answer to our first research question. Up until 2020, digital health and telehealth training was not present in any bachelor level degrees for health students in France. The SaNuRN initiative was launched in 2022 as a national development to fill this gap (42). SaNuRN is translated as “Digital Health Rouen-Nice” in English. It a project spanning 5 years via a partnership between the University of Rouen Normandy (URN) and Côte d’Azur University (CAU) who have established a consortium to enhance digital and telehealth education for all medical, paramedical, health practitioners, students, and administrators. Development is based on the *French National Referential on Digital Health* (*FNRDH*), defining the competencies and skills which must be to be gained and validated for health practitioners and every student in health, paramedical, and social services programs (42). The project has developed instructional unit, educational resources, a skills framework and implementation guidelines. The program is delivered via an online platform which is undergoing continuous innovation in telemedicine training, virtual interactive activities and a 3 h personalized training package cover all 70 *FNRDH* competencies (42). The initiative profiles the only nation-wide digital health and telehealth initiative identified in this search. It provides example of one nation's effort to ensure that the entire health workforce is familiarized with the fundamentals of telehealth practice and broader digital health developments.

Our review identified efforts across the medical profession to prepare either the current or future workforce for the realities of telehealth practice by embedding activities in existing courses in either undergraduate or registrar level training (43–53), however, in these cases the activities did not constitute major element of the curricula. The ability to embed more substantial components into existing curricula appears to be constrained, among other things, by the reality that substantial or major curricula changes may trigger whole of curriculum accreditation renewal process (65). Development may also be slow as telehealth competencies are not yet emphasised by most accrediting authorities. Encouragingly, one study (51) described a multi-institutional feasibility assessment and telehealth curriculum development project that included a specific module relating to the telehealth ‘clinical encounter’ (51), while Grosjean et al. (2024) reported efforts in France to develop a training program related to the universal French National Referential on Digital Health (FNRDH).

**Figure 4:**
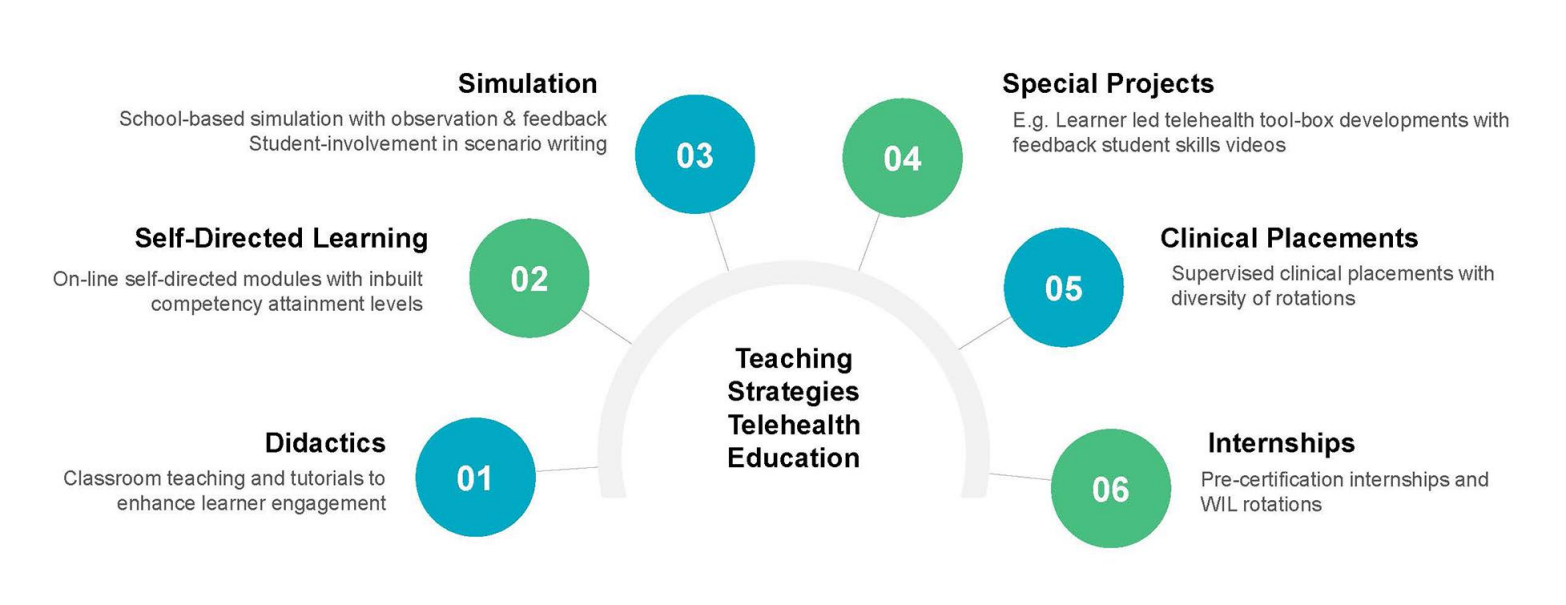
Educational strategies to teach telehealth competencies Concepts drawn from (26, 55).

In a nursing context, our review identified works which primarily addressed the needs of experienced nurses in either speciality, semi-autonomous primary care or nurse practitioner roles (26, 41, 54–56). These works utilized a variety of teaching strategies to develop telehealth competencies are useful in facilitating discussions to improve perceptions regarding possible teaching strategies and PEOU. Figure 4 details the range of teaching strategies used within the educational initiatives of Bobek and George et al. Educational strategies included didactic instruction, simulation, self-directed learning, internships and practice placements (26, 55). The necessity of practice-based placements appeared to be particularly important as emphasized earlier by van Houwelingen, something which nursing regulatory bodies have been slow to either recognize or approve (1).

Much of the curricula materials or program guidelines discovered in this review focus primarily on the technological aspects of service delivery such as computer literacy, information literacy and information management, rather than telehealth care delivery (66–68). While the National League of Nursing (NLN) provided a vision for preparing nursing students for technology, the focus was on electronic health records (EHR) and informatics, rather than telehealth for the provision of care (69). Caution regarding an overemphasis on the ‘’technology’ vs. ‘practice’ and ‘care’ aspects of telehealth guidelines and lack of integration into education programs appears to be longstanding (18, 67). It is unarguable that to practice telehealth safely, healthcare professionals (including nurses) need to be competent in the use of telecommunications and digital devices, however, they must also receive the clinical skills training to ensure safe practice during a telehealth encounter (18, 24).

### Research question 2: telehealth competencies and safe practice

Evidence pointing to the positive impact of nursing related telehealth services in, for example, reductions in presentation to acute care services (70) cements the reality that telehealth will continue to grow. As development proceeds, patient safety remains an ongoing concern (71–74). Of concern, with the exception of the 70 nationally agreed competencies with the French SaNuRN initiative (42), this search failed to locate literature detailing any clear, comprehensive and universally agreed national, global or profession specific curricula or competencies for telehealth practice, particularly competencies related to safe practice in telehealth care. Competencies needed for safe practice were located within each of the selected works (see Table 3) but these were stand-alone pieces rather than nationally or globally agreed competencies or educational recommendations. This finding is consistent with a recent scoping review related to clinical guidelines to safely inform safe practices in virtual health assessment which also failed to locate professionally agreed standards for assessment during telehealth encounters (75). Clear standards, education, training and competency acquisition are essential for risk reduction in virtual triage and care (73, 76, 77).

Peer-reviewed literature is often constrained by journal word counts; therefore, detailed competency descriptors and curricula guidelines are more likely to appear within textbooks, ISBN reports and grey literature. Documents of this nature are beginning to appear across grey literature published by either a professional bodies or health workforce agencies and are more likely to have received formal professional endorsement (41, 50, 58–60).

Many large-scale call centres, virtual emergency departments and ambulance services are now using detailed software-based clinical pathways such as the Schmitt-Thompson Nurse-Telehealth Triage Guidelines (78), or in Australia Ambulance Victoria's Secondary Triage Guidelines (79) to guide nursing responses during calls. Whilst effective, algorithms alone are insufficient to avoid risk. Nurses must also have the alertness and critical thinking competencies to detect cues that may be missed by following an algorithm as the only input to clinical decision making. Critical thinking, clinical decision making and risk assessment competencies are also needed in relation to which clients are suitable for telehealth consults and where for safety reasons, face-to-face consult may be required.

Competencies distilled during the data analysis aspect of this search (see Table 3) are mapped across a logical sequence of a call encounter and what could be described as ‘call management’ competencies related to call control and time management competencies. However, specific competencies related to call management, call control and time management were not identified within the literature. Discussion with practice partners supporting this research spoke to the tremendous pace of practice change which has embedded telehealth practice as now routine. In a virtual care context such as the Victorian Virtual Emergency Department located in Australia (80), it is common for telehealth based nurses to manage up to 40 calls per shift in the VVED context, thus time management and call control skills are a critical competency.

Beyond work to identify and agree the critical competencies for effective and safe telehealth, the need for these competencies needs to be understood by nurse educators and be reflected in course content, design and clinical placements. Educators must also consider the different educational approaches needed in respect to telephone vs. video or digital device- based consults (43).

### Research question 3: implications for nursing education

Nurses increasingly hold a pivotal role in facilitating and delivering telehealth services but many engage in telehealth practice with little or no formal training (21). This review has confirmed the woeful 45-year academic-practice gap and the reality that nursing education providers have been slow in responding to changes in the practice environment. Responses of nursing regulators have been equally slow with little movement in mandating clinically orientated telehealth competency requirements within regulatory and accreditation standards. Most nursing education programs do not prepare nurses for practice in either telehealth triage, virtual clinical health assessment, or telehealth encounters (5). A national US-based study reported a low level of telehealth content and experience across nursing education programs with 55% of prelicensure and 40% of graduate programs having no telehealth related content. The top barriers were reported as lack of funds, faculty experience and faculty buy-in (12). Thus, 21st century graduates often find themselves in a vulnerable position of transitioning to the workforce without the required skills and competencies (18).

Livesay et al. (2024), report a study involving a gap analysis between competency requirements for digital health practice and what is found within nursing education curricula. Again, the focus is on the broader aspect of digital health and relatively silent on clinically focussed telehealth practice. Nevertheless, the authors report significant gaps between the requirements and realities of practice environments and what are presently taught within nursing curricula (81). These authors highlight that while some nurse educators are keen to acquire these skills, some are resistant, and many confess a lack of knowledge or the confidence to teach (82). Other research has also stressed the need for greater focus on closing the current academic/practice gaps (12, 68, 81) and for early adopting nurse educators to be better recognized, encouraged and supported (83). And, of course, nurse educators require the support of professional regulatory bodies to recognize the trends in telehealth practice and formally reflect this within program standards, accreditation criteria and clinical placement provisions.

### Further research: understanding the education-practice Gap

The findings in this review raise question regarding possible reasons for the longstanding education practice gap which is evident in this field. The *Technology Acceptance Model* (TAM) is a useful aid to help understand the situation (84, 85). TAM is a theory which sheds light on how new technology is accepted and utilized by users. TAM has been used to explore acceptance of new e-technology and e-services by focussing on two main beliefs, specifically ‘perceived usefulness (PU) and ‘perceived ease of use’ (PEOU) and their relationship as key drivers of ‘attitudes towards use’ (ATU) and ‘behavioural intention to use’ (BI). The theory was developed in the late 1980s by Fred Davis (86) extending the earlier work of Martin Fishbein and Icek Ajzen and the *Theory of Reasoned Action* (87). Collectively, these works aim to understand and explain the relationships between human perceptions, attitudes, and actions. Applied to this context, they relate to ATU and BI associated with accepting, adopting, regulating, and teaching safe practice in virtual care.

While the scope of this review does not explore the reasons for the education–practice gap and/or the perceptions and beliefs that may underlie the lag in uptake, it is clear that more needs to be done to allay fear, change beliefs, and alter attitudes regarding both the perceived usefulness (PU) and perceived ease of use (PEOU) of digital innovation and virtual care. If teaching faculty fear the unknown and lack confidence to teach, this will negatively impact attitudes toward use (ATU) and behavioural intention (BI), and the education–practice gap will continue to exist. Similarly, unless regulators embrace the reality of the rapidly changing practice landscape, adopt positive ATU, and act with purpose (BI) to embed virtual care within mandated educational requirements, closure of the education–practice gap will continue to lag. Intentional strategy and support are needed to close education-practice gaps (88) improve the understanding of regulators and increase the confidence of educators to deliver content related to safe practice in virtual care. These requirements should be usefully informed by further research. The *Theory of Reasoned Action* (87) and the *TAM Technology Acceptance Model* (84, 85, 87) would provide useful theoretical frameworks for further work.

### Contributions of finding to the wider global community?

- Telehealth practice is increasingly common and enables better use of expertise across the health workforce, helping to address issues of health service access, particularly for rural, remote, and marginalized communities
- The safety of telehealth services is increased if delivered by appropriately qualified health professionals
- However, competence in face-to-face care provision does not automatically transfer to competency in telehealth delivery
- Our findings highlight a significant gap in respect to preparation of the current and future workforce for safe practice in virtual contexts.
- There is an **urgent** need to accelerate the inclusion of telehealth competencies into nursing and health workforce curricula and in-service professional development activities
- Intentional strategy and support is needed to close the current education-practice gaps in respect to understanding and education regarding safe practice in virtual care.

## Limitations

This review is limited to the search terms used to guide the search process. Different search teams may yield different results. Also, the review included studies in English only with non-English works excluded. A targeted grey literature search was undertaken; however, only a limited number of websites were able to be searched. Specifically, only the first 100 titles were read in detail for each of the located websites. In respect to grey literature, authors acknowledge that competency statements and curricula resources may also be being used internally within education or health systems, or informally amongst educators and not publicly accessible.

## Conclusion

Technical innovation and telehealth services are undergoing rapid expansion as key strategies in enhancing health care access. Telehealth services are a safe and effective modality if delivered by an appropriately qualified nurse or other health professional, however, competence in face-to-face care provision does not automatically transfer to competency in telehealth delivery. This search illustrates a slow uptake in embedding new practice models in nursing and other health professional programs. Need exists to confirm the competency requirements for safe practice and clinical competence in telehealth delivery. There is an imperative to accelerate related content into nursing curricula and professional development activities for both the existing and emerging health workforce. Support is needed to increase the confidence of the nursing education workforce in teaching content related to virtual care. The search identified a series of clinical competencies pertinent to telehealth-based clinical encounters, including competencies to prepare for a consult, communicate with clients; conduct the consult and close. These findings provide information for educators seeking clinically orientated guidance for telehealth inclusions in undergraduate nursing curricula and nursing professional development programs.
