# Supplementary material for: Preparing the workforce for telehealth practice: a scoping review
Source: Front Health Serv. 2026 Mar 18;6:1807317. doi: 10.3389/frhs.2026.1807317 (PMC13039038; doi:10.3389/frhs.2026.1807317)
Supplement: Supplementary file 1 [file Table1.docx]

**Search Terms**

Medline (Ovid)

1. (Telehealth or tele-health or telehealthcare or tele-healthcare or telemedicine or tele-medicine or telemonitor* or tele-monitor* or telenurs* or tele-nurs* or telepractice* or tele-practice* or telecare or tele-care or telescreen* or tele-screen* or telerehab* or tele-rehab* or teleconsult* or tele-consult* or telepsyc* or tele-psyc* or teleconferenc* or Tele-conferenc* or teletreatment* or tele-treatment* or teleclinic* or tele-clinic* or Tele-triag* or Teletriag* or Televisit* or Tele-visit* or telemental or tele-mental).ti,ab,kf.
2. (Ehealth or e-health or ehealthcare or e-healthcare or Emedicine or e-medicine or e-monitor* or Enurs* or e-nurs* or Epractice* or e-practice* or Ecare or e-care or Escreen* or e-screen* or Erehab* or e-rehab* or Econsult* or e-consult* or Eclinic* or e-clinic or e-clinics or Etriag* or E-triag* or Eexam* or e-exam* or Evisit* or e-visit*).ti,ab,kf.
3. (Mhealth or m-health or Mhealthcare or m-healthcare or mobile health).ti,ab,kf.
4. ((virtual or online or remote or video or digital or telephone) adj (health or healthcare or nurs* or assessment* or consult*)).ti,ab,kf.
5. Telemedicine/ or telenursing/ or mental health teletherapy/ or telerehabilitation/ or remote consultation/ or digital health/
6. 1 or 2 or 3 or 4 or 5
7. (health assessment* or psychosocial assessment* or psycho-social assessment* or healthcare assessment* or nurs* assessment* or physical assessment* or triag* or physical exam* or health exam* or health check* or clinical exam* or preventative health or preventative healthcare or health screen* or patient assessment* or patient assisted assessment* or primary survey* or secondary survey* or first line care).mp.
8. triage/ or nursing assessment/ or "Referral and Consultation"/
9. 7 or 8
10. ((virtual or online or remote or video or digital or telephone or phone) adj2 (health assessment* or psychosocial assessment* or psycho-social assessment* or healthcare assessment* or nurs* assessment* or physical assessment* or triag* or physical exam* or health exam* or health check* or clinical exam* or preventative health or preventative healthcare or health screen* or patient assessment* or patient assisted assessment* or primary survey* or secondary survey* or first line care)).mp.
11. 6 and 9
12. 10 or 11
13. curricul*.ti,ab,kf,sh.
14. educat*.ti,ab,kf.
15. (learning outcome* or learning objective*).ti,ab,kf.
16. (Competency or competencies).ti,ab,kf.
17. (training adj (program* or method*)).ti,ab,kf.
18. Pedagog*.ti,ab,kf.
19. ((clinical or core) adj (training or skill*)).ti,ab,kf.
20. (teach or teaching).ti,ab,kf.
21. (academic adj (course* or subject* or program* or training)).ti,ab,kf.
22. education, nursing, graduate/ or exp education, nursing/ or Competency-Based Education/ or Students, Nursing/
23. 13 or 14 or 15 or 16 or 17 or 18 or 19 or 20 or 21 or 22
24. 12 and 23
25. limit 24 to english language
